# Exploring Weak Magnetic Signal Characteristics of Pipeline Welds: Insights into Stress Non-Uniformity Effects

**DOI:** 10.3390/s24155074

**Published:** 2024-08-05

**Authors:** Xiangfeng Fan, Lijian Yang

**Affiliations:** School of Information Science and Engineering, Shenyang University of Technology, Shenyang 110870, China; yanglijian888@163.com

**Keywords:** weak magnetic detection, stress non-uniformity, magnetic charge, weld

## Abstract

Weak magnetic detection technology can detect stress concentration areas in ferromagnetic materials. However, the stress non-uniform characteristics of pipeline welds lead to significant differences in stress distribution range and values between inner wall welds and outer wall welds. This discrepancy makes it crucial to further evaluate the impact of stress non-uniformity on magnetic signals. To study the magnetic signal characteristics under the influence of residual stress in weld seams, a magneto-mechanical analytical model was established based on the magnetic charge theory and the distribution characteristics of residual stress in the weld seam. The magneto-mechanical relationship and magnetic signal distribution characteristics at the inner and outer wall welds of the pipeline are calculated. Furthermore, the effects of different excitation intensities on the amplitude growth characteristics of magnetic signals are analyzed and compared. To verify the analysis model, weld detection experiments with different excitation intensities were designed. The results show that both the peak-to-valley values of the normal component and the peak values of the tangential component of the outer wall weld are lower than those of the inner wall weld. Conversely, the peak-to-valley width of the normal component and the peak width of the tangential component are greater than those of the inner wall weld. Additionally, the rate of increase in weak magnetic signal amplitude decreases in a first-order exponential relationship with increasing excitation intensity. The average decay rates of the normal and tangential component amplitude growth rates for the inner wall weld are 34.03% and 27.9%, respectively, while for the outer wall weld, they are 31.75% and 28.01%, respectively. This study contributes to the identification and quantitative assessment of weak magnetic signals in inner and outer wall welds.

## 1. Introduction

Petroleum and natural gas, as crucial strategic resources for the nation, are intimately tied not only to the economic livelihood of its citizens but also play a paramount role in national military and defense security. Hence, both domestically and internationally, there is significant emphasis placed on ensuring the secure operation of long-distance oil and gas pipelines [1]. In the process of storage and transportation, petroleum and natural gas carry inherent risks of flammability and explosiveness, underscoring the critical importance of pipeline integrity management and safety maintenance [2,3]. Weld seams, as crucial components in pipeline connections, are susceptible to increased failure probability due to inherent residual stresses and micro-damages compounded by external factors such as geological movements [4,5]. The failure of weld seams further results in a series of adverse consequences, including oil and gas leaks, pipeline shutdowns, and other detrimental impacts [6,7].

Currently, magnetic flux leakage (MFL) detection technology stands as one of the most widely utilized methods in the field of pipeline internal inspection [8,9]. Magnetic inspection technology mainly consists of magnetic flux leakage detection and weak magnetic field detection [10]. Although magnetic flux leakage (MFL) detection technology enables internal inspection of pipelines with high precision and interference resistance, it is primarily sensitive to macroscopic defects and cannot effectively assess microscopic damages or stress concentration areas in ferromagnetic materials [11].

Weak magnetic field detection is an emerging, non-destructive testing technology. Its ability to precisely detect stress concentrations and provide early diagnosis and warnings for ferromagnetic materials has made it widely applicable in the field of micro-damage detection in long-distance oil and gas pipelines [12,13,14]. In the realm of weak magnetic detection in pipelines, Aroba Saleem proposed that pipeline failure may stem from the presence of stress concentration zones internally and externally, leading to corrosion-induced cracking failure. Employing weak magnetic detection technology enables non-contact, dynamic detection of micro-damage in buried pipelines [15]. J. Jesús Villegas-Saucillo developed a weak pipeline magnetic signal measurement system comprising high-resolution magnetic reluctance sensors and a data processing system. This system can monitor the state of ferromagnetic structural defects in real-time by analyzing the spontaneous leakage magnetic field distribution around pipeline defects [16]. R. Größinger studied the microstructure, properties, and magnetostrictive performance of retired pipelines, demonstrating that the pipelines exhibit magnetic anisotropy with the easy magnetization axis aligned along the axial direction of the pipeline [17]. Lijian Yang, based on the J-A model, established a pipeline magneto-mechanical coupling model using molecular current theory. Combining this with the magnetization curve of ferromagnetic materials under non-hysteresis conditions, the model was utilized to analyze and calculate the influence of external magnetic field and stress on the magnetic field on the surface of the pipeline wall along the magnetization direction [18].

Due to the weld welding process, the signal at the weld is relatively complex, and experts and scholars at home and abroad have carried out a lot of research work on the problem of weld detection. Bin Liu analyzed the weak magnetic signals of pipe welds, weld cracks, and metal losses of pipe base materials under different pressures and analyzed the propagation characteristics of metal magnetic memory signals in different media [19]. Guoxi He proposed a new parameter that reflects the stress state of a girth weld [20]. Wenzhi Wang described the weld cracks by the uniform magnetic field distortion (UMFD) method, established an analytical model of isolated cracks, and evaluated the location and width of the cracks [21]. Due to the complex stress distribution characteristics of welds, there is no perfect solution for the stress detection of girth welds.

In the absence of macroscopic cracks, the weak magnetic signal at the weld seam is primarily influenced by residual stress. Welding conditions, in turn, affect the formation and distribution of residual stress [22,23], resulting in a non-uniform distribution along the weld seam. Particularly, significant differences exist in the range and magnitude of stress distribution between the inner wall (back of the weld) and outer wall (face of the weld) weld seams of the pipeline.

Considering the influence of stress distribution on the inner and outer wall welds on the quantitative calculation of magnetomechanics, this paper first analyzes the stress distribution characteristics of the weld from a thermodynamic perspective and then quantitatively calculates the magneto-mechanical relationship at the inner and outer wall welds based on magnetic charge theory. The growth characteristics of the weak magnetic signal amplitude with increasing excitation intensity are analyzed and compared. In addition, systematic experimental research has been conducted to verify the feasibility of the proposed method, providing a scientific basis for the application of weak magnetic detection in the field of weld inspection in long-distance oil and gas pipelines.

## 2. Mathematical Model Establishment

**Figure 1:**
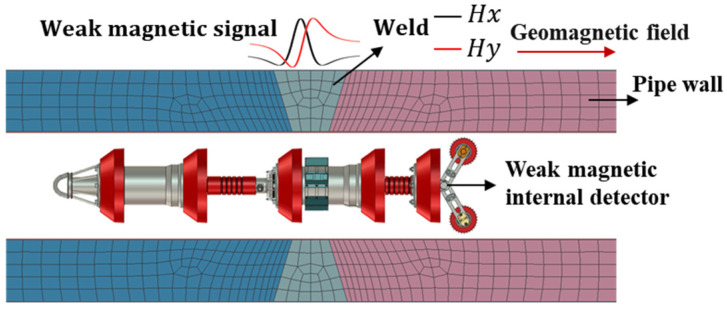
Schematic diagram of weak magnetic field internal detection in pipelines.

During pipeline welding, non-uniform temperature conditions and thermal expansion and contraction lead to the formation of residual stress in the weld seam and its surrounding areas [24]. In the stress concentration area, the magnetic domains turn along the easy magnetization axis, causing the accumulation of magnetic charges, thereby forming new magnetic poles at both ends of the stress concentration area [25,26,27]. Macroscopically, it manifests itself as a sudden change in the self-leakage magnetic field around the weld [28,29], as shown in Figure 1.

The stress effect can be equivalent to an additional magnetic field [30], and the equivalent magnetic field H combined with the geomagnetic field can be expressed as [31]:

(1) $$H_{e}=H+\alpha M+H\sigma =H+\alpha M+\frac{3\sigma }{2\mu_{0}}\left(\frac{d\lambda }{dM}\right)$$

In the formula, H is the intensity of the geomagnetic field, α is the magnetization coupling coefficient, M is the magnetization intensity, σ is the stress, $\mu_{0}$ is the vacuum permeability, and λ is the magnetostriction coefficient. The relationship between the magnetostriction coefficient of the material and the magnetization intensity can be expressed as:

(2) $$\lambda =\left(\gamma_{1}(0)+\gamma_{1}^{\prime }(0)\sigma \right)M^{2}+\left(\gamma_{2}(0)+\gamma_{2}^{\prime }(0)\sigma \right)M^{4}$$

In the formula, $\gamma_{1}$, $\gamma_{1}^{\prime }$, $\gamma_{2}$, and $\gamma_{2}^{\prime }$ are constants, which are related to the material. Substituting Equation (2) into Equation (1) and sorting it out, we can obtain:

(3) $$H_{e}=H+\alpha M+\frac{3\sigma }{2\mu_{0}}\left(\left(2\gamma_{1}(0)+2\gamma_{1}^{\prime }(0)\sigma \right)M+\left(4\gamma_{2}(0)+4\gamma_{2}^{\prime }(0)\sigma \right)M^{3}\right)$$

Assuming that the irreversible magnetization $M_{irr}$ obeys the proximity law, then:

(4) $$\frac{dM_{irr}}{dW}=\frac{1}{\xi }\left(M_{an}-M_{irr}\right)$$

In the formula, $M_{irr}$ is the irreversible component of magnetization, $M_{an}$ is hysteresis-free magnetization, and ξ is a constant related to the energy per unit volume. Among them, the hysteresis-free magnetization $M_{an}$ is expressed as [32]:

(5) $$M_{an}=M_{s}(\coth(\frac{H_{e}}{a})-\frac{a}{H_{e}})$$

In the formula, $M_{s}$ is the saturation magnetization, and a is the shape coefficient. According to the principle of minimum ability, the derivative of magnetization with respect to stress energy is expressed as:

(6) $$\frac{dM}{dW}=\frac{\left(1-c\right)}{\xi }\left(M_{an}-M_{irr}\right)+c\frac{dM_{an}}{dW}$$

In the formula, W is the magnetoelastic energy of unit volume stress, and its derivative of stress energy dW is:

(7) $$dW=(\frac{\sigma }{E})d\sigma$$

Substituting Equation (7) into Equation (6), we can obtain the relationship between magnetization intensity M and stress σ:

(8) $$\frac{dM}{d\sigma }=\frac{1}{\varepsilon^{2}}\sigma (1-c)(M_{an}-M_{irr})+c\frac{dM_{an}}{d\sigma }$$

In the formula, c reflects the flexibility coefficient of the magnetic domain wall, and ε is the coefficient related to stress. Substituting Equations (3)–(5) into Equation (8) and sorting out the relationship model between stress and magnetization intensity can be obtained as:

(9) $$\frac{dM}{d\sigma }=\frac{\frac{\sigma }{E\xi }\left(M_{an}-M\right)+cM_{s}\left[{\mathrm{csch}}^{2}\left(\frac{H_{e}}{a}\right)-\frac{a}{H_{e}^{2}}\right]\left\{\frac{3}{\mu_{0}}\left[\left(\gamma_{1}+\gamma_{1}^{\prime }\sigma \right)M+2\left(\gamma_{2}+\gamma_{2}^{\prime }\sigma \right)M^{3}\right]\right\}}{1-cM_{s}\left[{\mathrm{csch}}^{2}\left(\frac{H_{e}}{a}\right)-\frac{a}{H_{e}^{2}}\right]\left[\frac{3\sigma }{\mu_{0}}\left(\gamma_{1}+6\gamma_{2}M^{2}\right)+\alpha \right]}$$

**Figure 2:**
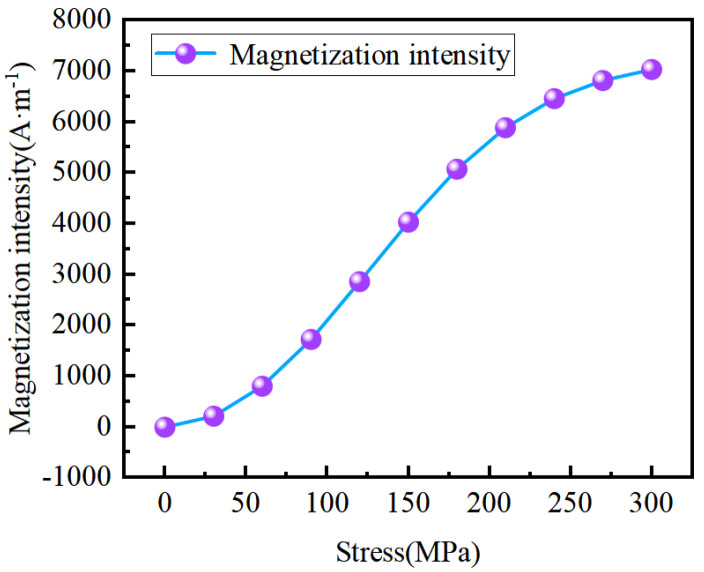
Stress magnetization curve.

The force-magnetic coupling relationship of ferromagnetic materials under weak magnetic fields is formed through the above formula to consider the influence of stress changes on the magnetization intensity of materials under constant weak magnetic fields. The material parameters are c=0.25, $\mu_{0}=4\pi \cdot 10^{-7}\cdot NA^{-2}$, $\gamma_{1}=7\cdot 10^{-18}\cdot A^{-2}\cdot m^{2}$, $\gamma_{1}^{\prime }=-1\cdot 10^{-25}\cdot A^{-2}\cdot m^{2}\cdot Pa^{-1}$, $\gamma_{2}=-3.3\cdot 10^{-30}\cdot A^{-4}\cdot m^{4}$, $\gamma_{2}^{\prime }=2.1\cdot 10^{-38}\cdot A^{-4}\cdot m^{4}\cdot Pa^{-1}$, and the saturation magnetization intensity $M_{s}=1.585\cdot 10^{6}A\cdot m^{-1}$ [33]; the stress magnetization curve can be calculated as shown in Figure 2.

**Figure 3:**
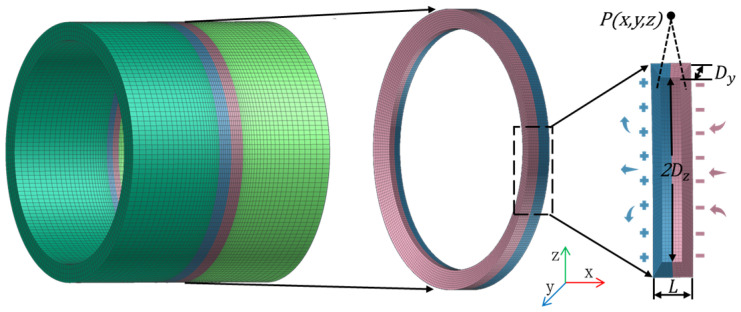
Three-dimensional schematic of magnetic charge model.

As shown in Figure 3, in the area of the pipeline weld seam, the occurrence of residual stress results in a polarization effect, wherein the magnetic permeability of the material decreases at the weld seam. This leads to the accumulation of magnetic charges and the formation of new magnetic poles at both ends of the weld seam.

According to the magnetic charge model illustrated in Figure 3, half of the magnetic charge distributed on the defect is positive and the other half is negative. The axes of coordinates *x*, *y*, and *z* are established along the three orthogonal directions of the cylindrical slot, and the length, width, and depth of the weld are *L*, 2*Dz*, and *Dy*, respectively. Assuming that the external magnetic field *H* is along the *X*-axis direction, the coordinates of the three-dimensional spatial field point are defined as P(*x*, *y*, *z*), and the coordinates of the source point of the magnetic charge surface are (*xm*, *ym*, *zm*), the magnetic field intensity generated by the microfacet element dymdzm on the magnetic charge surface at point P can be expressed as [34,35]:

(10) $$dH=\frac{\rho dy_{m}dz_{m}}{4\pi \mu_{0}r^{3}}\vec{r}$$

where $\vec{r}$ is the directional vector from the magnetic charge surface to the detection location,$\vec{r}$ is the distance and µ_0_ is the vacuum permeability, ρ is the magnetic charge density. Then, the leakage magnetic field signal formed by the two sidewalls of the weld seam at P can be obtained by integrating Equation (10) [36].

According to the magnetic charge model illustrated in Figure 3 and in conjunction with the magnetic dipole model, when stress exists at the weld seam, the magnetic signals $H_{x}$ and $H_{y}$ due to the polarization effect can be, respectively, represented as:

(11) $$H_{x}=\frac{\rho }{4\pi \mu_{0}}\int_{-D_{z}}^{D_{z}}\int_{-D_{y}}^{0}\frac{x+L/2}{{\left[{\left(x+L/2\right)}^{2}+{\left(y-y_{m}\right)}^{2}+{\left(z-z_{m}\right)}^{2}\right]}^{\frac{3}{2}}}-\frac{x-L/2}{{\left[{\left(x-L/2\right)}^{2}+{\left(y-y_{m}\right)}^{2}+{\left(z-z_{m}\right)}^{2}\right]}^{\frac{3}{2}}}dy_{m}dz_{m}$$

(12) $$H_{y}=\frac{\rho }{4\pi \mu_{0}}\int_{-D_{z}}^{D_{z}}\int_{-D_{y}}^{0}\frac{y-y_{m}}{{\left[{\left(x+L/2\right)}^{2}+{\left(y-y_{m}\right)}^{2}+{\left(z-z_{m}\right)}^{2}\right]}^{\frac{3}{2}}}-\frac{y-y_{m}}{{\left[{\left(x-L/2\right)}^{2}+{\left(y-y_{m}\right)}^{2}+{\left(z-z_{m}\right)}^{2}\right]}^{\frac{3}{2}}}dy_{m}dz_{m}$$

Among them, $H_{x}$ and $H_{y}$ are, respectively, the tangential and normal components of the magnetic signal at point P(x,y,z) near the specimen surface when there is magnetic field leakage in the material, and L is the axial width of the weld. ρ(x) represents the magnetic charge density. According to the magnetic charge theory, the magnetic charge density satisfies $\rho (x)=\mu_{0}M(\sigma ,H_{0})$.

## 3. Weld Model Creation

**Figure 4:**
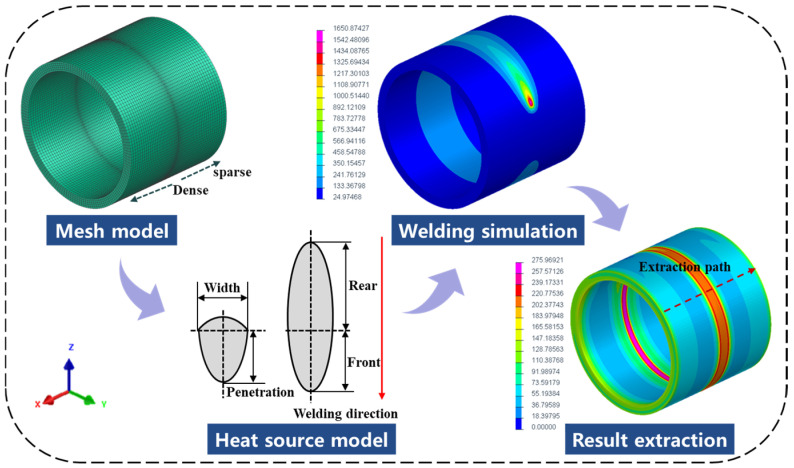
Pipeline weld simulation model.

During the welding process of the pipeline, the metal in the melting zone of the weld and the nearby area undergoes instantaneous high temperature and rapid cooling, and the microstructure of the weld material undergoes a phase change, causing changes in the material’s magnetic properties (magnetization intensity, magnetic permeability, etc.), resulting in the spatial magnetic field in the weld area producing distortion [37,38]. Therefore, based on determining the size and dimensions of the pipeline for establishing the model, a finite element simulation model is established. The thermodynamic properties of the model are the characteristics of pipeline material X70, and a suitable heat source model is used to simulate the welding interface. Finally, the analysis results are obtained, and the process is shown in Figure 4.

The temperature changes rapidly during the material welding process, so the division of the mesh will have a direct impact on the calculation and analysis results. Due to the large temperature gradient in the weld area and the complex stress and strain changes, in order to ensure the accuracy of numerical calculations, this paper adopts a fine grid division for the weld joint area and uses a coarser grid for the base metal part far away from the weld. Moreover, the grid adopts more hexahedral elements and as few tetrahedral elements as possible to avoid the problem of non-convergence of the function.

For the material properties in the simulation model, high-strength low-alloy (HSLA) pipeline steels are commonly chosen. Materials such as X70 and X80, commonly found in long-distance oil and gas pipelines, belong to the category of high-strength, low-alloy pipeline steels. The basic material properties of X70 steel are shown in Table 1.

**Figure 5:**
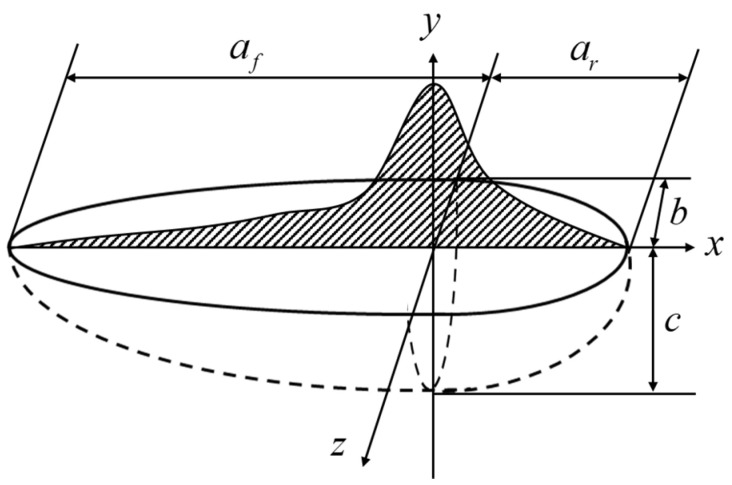
Double-ellipsoid heat source model.

The pipeline welding process parameters are as follows: The weld groove shape is V-shaped, the welding method is submerged arc welding, the welding voltage (U) is 25 V, the welding current (I) is 200 A, the welding speed (V) is 5 mm/s, and the ambient temperature (T) is 25 °C. A double-ellipsoid heat source model is used [40], as depicted in Figure 5.

The heat source model satisfies the following equation:

(13) $$q_{f}(x,y,z)=\frac{6\sqrt{3}f_{f}Q}{a_{f}bc\Pi \sqrt{\Pi }}\exp(\frac{3x^{2}}{a_{f}^{2}}+\frac{3y^{2}}{b^{2}}+\frac{3z^{2}}{c^{2}}),x\geq 0$$

(14) $$q_{r}(x,y,z)=\frac{6\sqrt{3}f_{r}Q}{a_{r}bc\Pi \sqrt{\Pi }}\exp(\frac{3x^{2}}{a_{r}^{2}}+\frac{3y^{2}}{b^{2}}+\frac{3z^{2}}{c^{2}}),x<0$$

In the formula, q is the heat flux, $J/(m^{2}\cdot s)$; x,y,z are the coordinates relative to the center of the heat source; $a_{f}$ is the front length of the ellipsoid, mm; $a_{r}$ is the length after the ellipsoid, mm; b is half the width of the ellipsoid, mm; c is the depth of the ellipsoid, mm; Q is the ellipsoid depth and is the effective power, W; where Q is calculated as follows:

(15) $$Q=\frac{I\times U\times \eta }{V}$$

Among them, η is the arc thermal efficiency; U is the welding voltage, V; I is the welding current, A.

**Figure 6:**
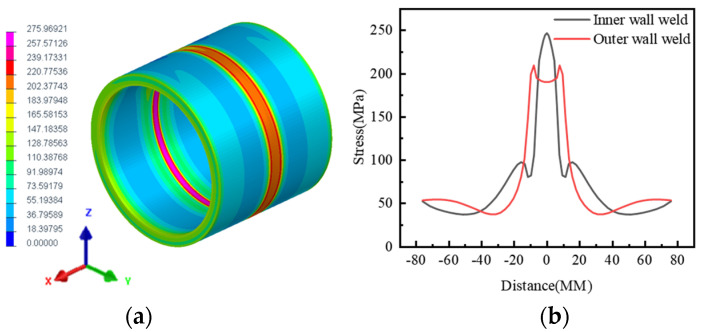
Residual Stress on Inner and Outer Surfaces of Weld Seam (**a**) Stress Distribution Cloud Map (**b**) Stress Distribution Curve.

The structural boundary conditions adopt full constraints at both ends to prevent rigid displacement of the model during the calculation process. The temperature field condition is heat exchange with air, and the ambient temperature is 25 °C. According to the established pipeline model and set process parameters, the residual stress size and distribution after welding can be calculated. This study mainly studies the changes in the weak magnetic field caused by Von Mises stress. The Von Mises stress distribution is shown in Figure 6.

As depicted in the above figure, the peak residual stress of the inner wall weld is 247 MPa, with a stress concentration range of approximately 24 mm. Meanwhile, the peak residual stress of the outer wall weld is observed in the weld fusion zone, reaching 210 MPa. Additionally, the peak residual stress in the center of the weld measures 200 MPa, with a stress concentration range of about 30 mm. It is evident that while the residual stress amplitude of the inner wall weld is larger, the stress concentration range is smaller, whereas the residual stress amplitude of the outer wall weld is smaller, but the stress concentration range is larger.

## 4. Magneto-Mechanical Relationship Calculation

**Figure 7:**
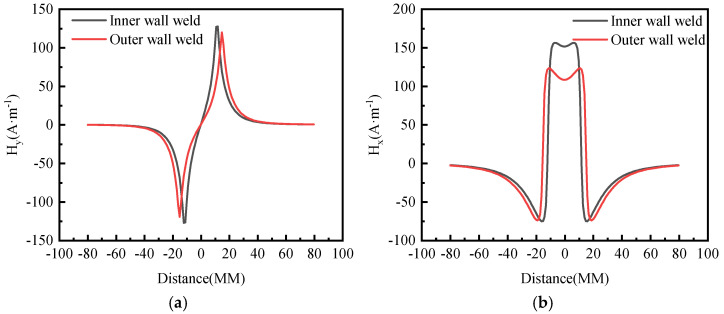
Weld Seam Inner and Outer Wall Weak Magnetic Signals (**a**) Normal Component (**b**) Tangential Component.

Based on the magnetic charge theory and the simulation results of weld stress, the weak magnetic signal of the weld on the inner and outer walls of the pipeline is calculated, as depicted in Figure 7.

From the calculation results, it can be observed that the normal component $H_{y}$ of the weak magnetic signal crosses the zero point and fluctuates sinusoidally, while the tangential component $H_{x}$ exhibits extreme values. This is due to the influence of high temperatures during the welding process, which causes metal phase transformation at the weld seam and changes in stress, resulting in a change in the magnetic signal at the weld seam. At the same time, due to the trapezoidal distribution of the weld seam as a whole, there is a difference between the inner-wall signal and the outer-wall signal.

### 4.1. Weak Magnetic Signals in Inner Wall Welds

**Figure 8:**
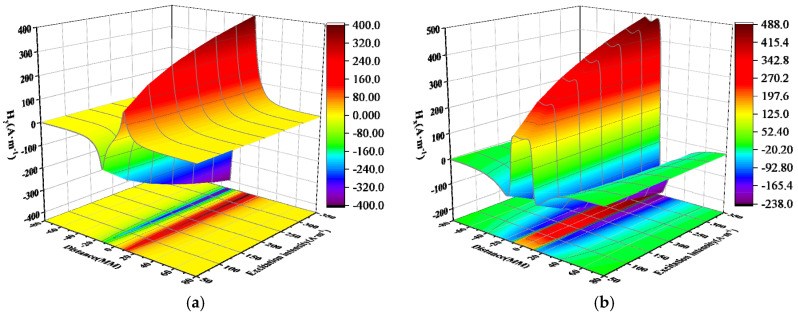
Weak Magnetic Signals of Inner Wall Welds (**a**) Normal Component (**b**) Tangential Component.

To study the characteristics of the weak magnetic signal on the inner wall of the pipeline weld, the center of the weld is selected as the coordinate origin, and a detection path of −80 to 80 mm along the pipeline axial direction is utilized for weak magnetic signal detection. The lift-off value is set to 1 mm, and the excitation intensity ranges from 50 to 350 A/m with an interval of 50 A/m. MATLAB20 software is employed to calculate the weak magnetic signal, as depicted in Figure 8.

As depicted in Figure 8, with the increase in the intensity of the external magnetic field, the peak-to-valley values of the normal component and the peak value of the tangential component of the weak magnetic signal increase. However, the peak-to-valley width of the normal component and the peak width of the tangential component remain unchanged. The spacing, which corresponds to the stress concentration width of the inner wall weld, is 24 mm.

**Figure 9:**
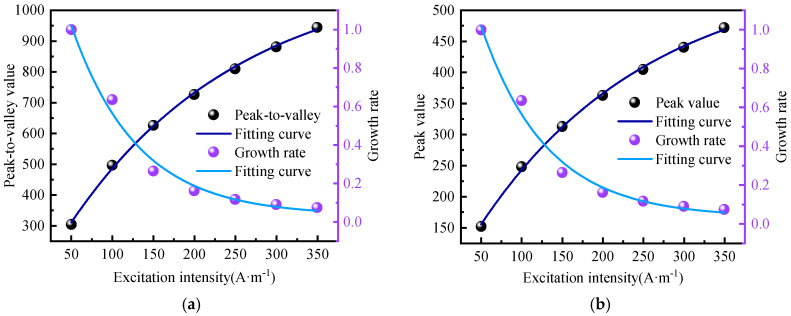
Rate of Amplitude Growth (**a**) Normal Component (**b**) Tangential Component.

To investigate the specific changing characteristics of the weak magnetic signal of the inner wall weld under different excitation strengths, the peak-to-valley values of the normal component and the peak value of the tangential component were extracted, and their amplitude growth rates were calculated. The calculation results are presented in Figure 9.

From Figure 9, it is evident that the growth rates of the normal component and the tangential component follow the same trend. Under an excitation intensity of 50 to 250 A/m, the signal amplitude growth rate decays rapidly. When the excitation intensity increases to 250 A/m, the signal amplitude growth rate slows down to 10%. With further increases in excitation intensity, the signal amplitude growth rate gradually approaches 0, demonstrating a first-order exponential decline relationship. The average attenuation rate is 15.47%, which can be approximated by the following relational expression:

(16) $$y=0.032+1.834e^{-x/80.649}$$

### 4.2. Weak Magnetic Signals in Outer Wall Welds

**Figure 10:**
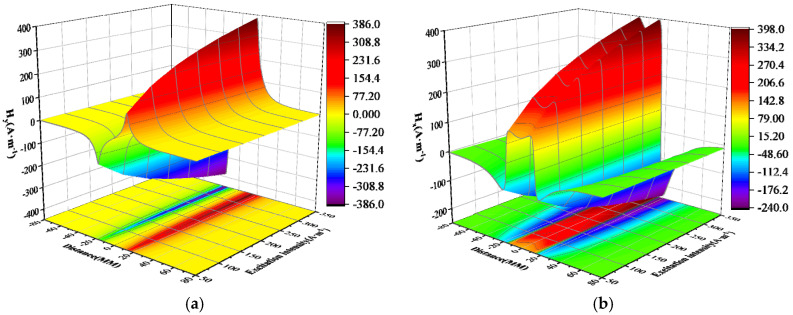
Weak Magnetic Signals of Outer Wall Welds (**a**) Normal Component (**b**) Tangential Component.

To study the characteristics of the weak magnetic signal on the outer wall of the pipeline weld, the center of the weld is chosen as the coordinate origin, and a detection path of −80 to 80 mm along the pipeline axial direction is employed for weak magnetic signal detection. The lift-off value is set to 1 mm, and the excitation intensity ranges from 50 to 350 A/m with an interval of 50 A/m. MATLAB software is utilized to calculate the weak magnetic signal, as depicted in Figure 10.

As depicted in Figure 10, with the increase in the intensity of the external magnetic field, the peak-to-valley values of the normal component and the peak value of the tangential component of the weak magnetic signal increase. However, the peak-to-valley width of the normal component and the peak width of the tangential component remain unchanged. The spacing, which corresponds to the stress concentration width of the outer wall weld, is 30 mm.

**Figure 11:**
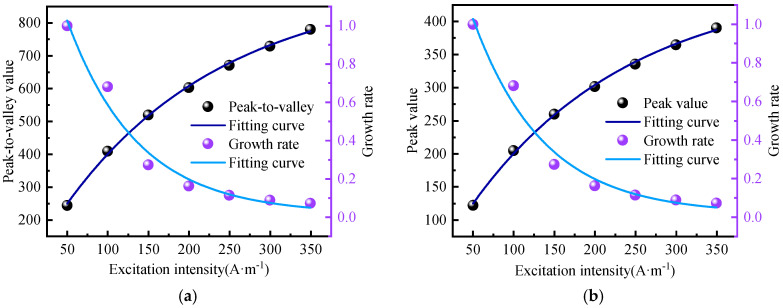
Rate of Amplitude Growth (**a**) Normal Component (**b**) Tangential Component.

To investigate the specific changing characteristics of the weak magnetic signal of the outer wall weld under different excitation strengths, the peak-to-valley values of the normal component and the peak value of the tangential component were extracted, and their growth rates were calculated. The calculation results are presented in Figure 11.

From Figure 11, it is evident that the growth rates of the normal component and the tangential component exhibit the same trend. Under an excitation intensity of 50 to 250 A/m, the signal amplitude growth rate decays rapidly. When the excitation intensity increases to 250 A/m, the signal amplitude growth rate decreases to 10%. As the excitation intensity continues to increase, the signal amplitude growth rate gradually approaches 0, demonstrating a first-order exponential decline relationship. The average attenuation rate is 15.49%, which can be approximated by the following relational expression:

(17) $$y=0.0151+1.798e^{-x/87.168}$$

Combining Figure 8 and Figure 10, it is evident that under the same excitation intensity, the peak-to-valley values of the normal component and the peak value of the tangential component of the outer wall weld are both lower than those of the inner wall weld. However, the peak-to-valley width of the normal component and the peak width of the tangential component of the outer wall weld are both larger than those of the inner wall weld. Combining Figure 9 and Figure 11, it is evident that the excitation intensity has an influence on the amplitude of the weak magnetic signal, and this influence diminishes as the excitation intensity increases. Whether it is an inner wall weld or an outer wall weld, the relationship between the signal amplitude growth rate and the excitation intensity follows a first-order exponential decline relationship represented by $y=y_{0}+Ae^{-x/t_{1}}$.

## 5. Experimental Results and Analysis

To verify the correctness of the theoretical model, this paper designed welding seam detection experiments with varying excitation strengths.

### 5.1. Experimental Materials

**Figure 12:**
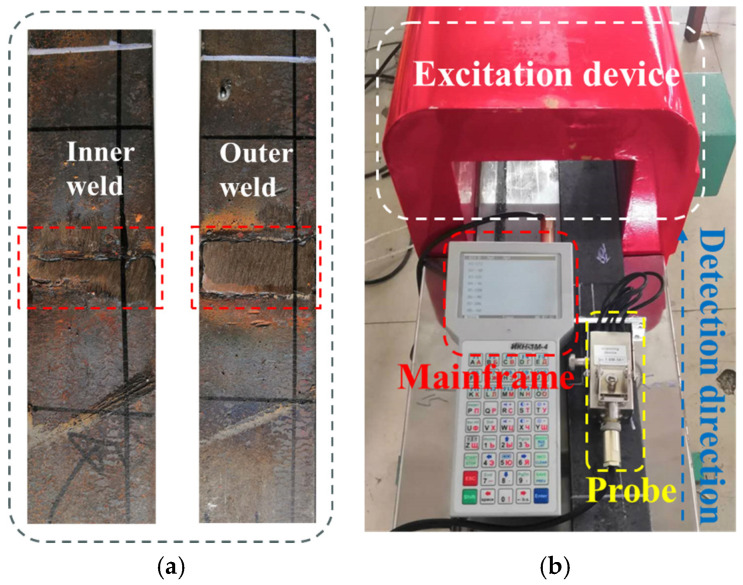
Detection Test Materials (**a**) Test Specimens (**b**) TSC-1M-4.

The X70 pipe was selected as the experimental material, and a rectangular specimen was cut from the axial direction of the pipe and pre-flattened. The experimental specimen is depicted in Figure 12a. The excitation device is an excitation coil; the outside is covered by a magnetic shielding shell, in which the number of turns of the experimental excitation coil is about 3931 turns, and the effective magnetic circuit length is about 1 m. The excitation current is obtained to be 0~31.2 kA/m for the excitation current from 0 A to 8 A. The external magnetic field strength is 0~31.2 kA/m. The magnetic signal detection equipment TSC-1M-4 was utilized for the experiments. This detection equipment collects weak magnetic signals through high-precision probes and stores the recorded data on the host computer, as illustrated in Figure 12b.

Put the equipment under test into the excitation device for magnetization, and then take it out for magnetic signal measurement. The fixed lift-off value is 1 MM. The excitation currents are 1.5 A, 2.5 A, 3.5 A, 5 A, and 7.5 A, respectively. The probe is gradually advanced along the width direction of the weld and records the weak magnetic signals at different positions.

### 5.2. Inner Wall Weld Magnetic Signal

**Figure 13:**
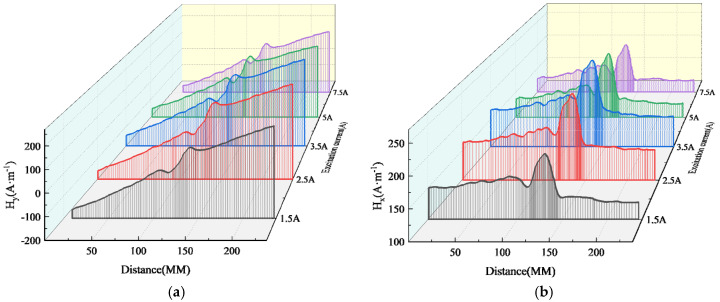
Inner Wall Weld Magnetic Signal (**a**) Normal Component (**b**) Tangential Component.

The experimental results of magnetic signal detection in the inner weld under different excitation strengths are shown in Figure 13. The horizontal axis represents the scanning path, while the vertical axis represents the spontaneously leaked magnetic field measured near the weld seam with a lift-off value of 1 mm. The measured normal and tangential components of the weak magnetic signal exhibit trends consistent with theoretical calculations.

**Figure 14:**
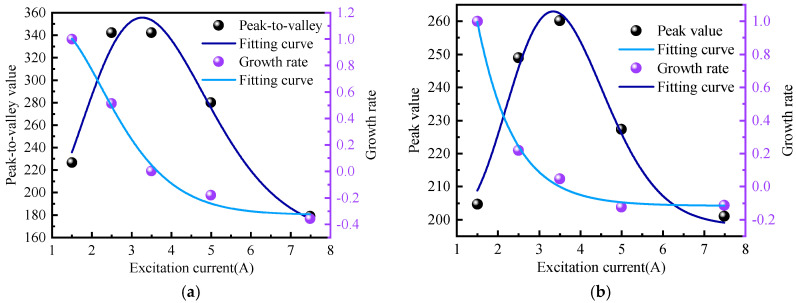
Rate of Amplitude Growth (**a**) Normal Component (**b**) Tangential Component.

It can be seen from the above figure that as the excitation current changes, the peak-to-valley width of the normal component of the weak magnetic signal and the peak width of the tangential component remain unchanged, and the distance between them is approximately 30 mm. However, the amplitudes of the normal component and the tangential component change with the change in the excitation current. The specific changes are shown in Figure 14.

It can be seen from the above figure that the amplitude growth rate of the weak magnetic signal of the inner wall weld shows a first-order exponential decline relationship with the excitation current. The average attenuation rate of the normal component amplitude growth rate is 34.03%, and the average attenuation rate of the tangential component amplitude growth rate is 27.9%. The attenuation rate is first fast and then slows down, which is the same as the theoretical calculation trend.

### 5.3. Outer Wall Weld Magnetic Signal

**Figure 15:**
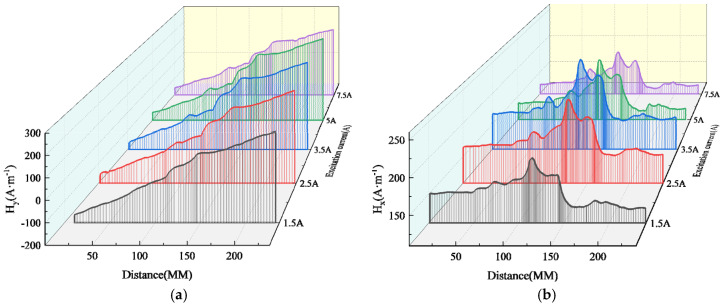
Outer Wall Weld Magnetic Signal (**a**) Normal Component (**b**) Tangential Component.

The experimental results of magnetic signal detection for the outer wall weld under different excitation strengths are presented in Figure 15. The observed weak magnetic signal exhibits a similar changing trend to that of the inner wall weld. However, under the same excitation intensity, the peak-to-valley value of the normal component and the peak value of the tangential component of the weak magnetic signal for the outer wall weld are lower than those of the inner wall weld. Additionally, the peak-to-valley width of the normal component and the peak width of the tangential component for the outer wall weld are both larger than those of the inner wall weld.

**Figure 16:**
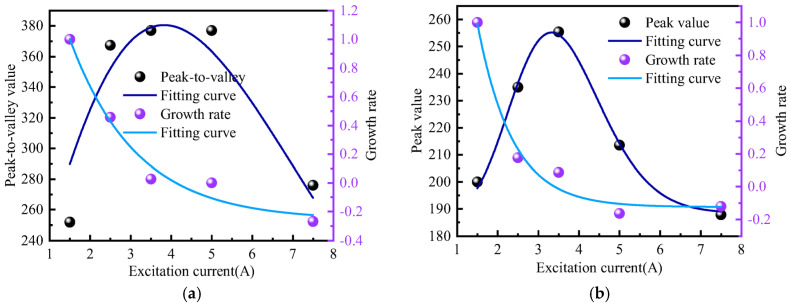
Rate of Amplitude Growth (**a**) Normal Component (**b**) Tangential Component.

It can be observed from the above figure that as the excitation current changes, the peak-to-valley width of the normal component and the peak width of the tangential component of the weak magnetic signal remain unchanged, with a spacing of approximately 40 mm. The growth rate of the normal component and tangential component amplitudes slows down as the excitation current increases. The specific changes are depicted in Figure 16.

It can be observed from the above figure that the growth rate of the amplitude of the weak magnetic signal for the outer wall weld exhibits a first-order exponential decline relationship with the excitation current. The average attenuation rate of the normal component amplitude growth rate is 31.75%, while the average attenuation rate of the tangential component amplitude growth rate is 28.01%. The attenuation rate initially decreases rapidly and then slows down, consistent with the theoretical calculation trend.

### 5.4. Comparison of Inner and Outer Wall Weld Magnetic Signal

According to the experimental result data, it can be seen from Figure 14 and Figure 15 that the variation width of the weak magnetic signal of the outer weld is larger than that of the inner weld, which is due to the abrupt change in the magnetic signal on the two end surfaces of the magnetic charge aggregation of the weld, which results in the correlation of the width of the signal with the width of the weld.

**Figure 17:**
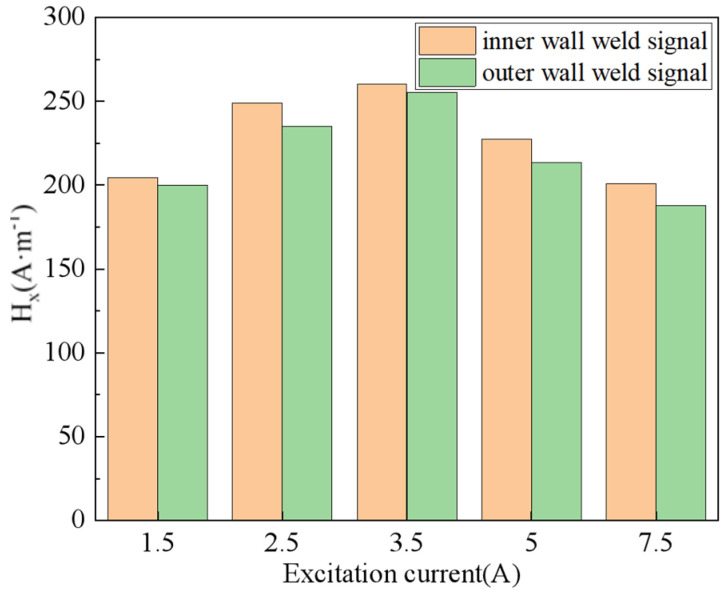
Comparison of Peak Values of Inner and Outer Wall Weld Magnetic Signal Signals.

In order to further compare the experimental data and analyze the characteristics of the weak magnetic signals of the inner and outer wall welds, the radial signals with more obvious signal peaks were extracted and plotted, as shown in Figure 17.

Through Figure 17, it can be noticed that the magnitude of the outer wall is greater than the inner wall magnitude. This is because the weld starts from the inner weld to the outer weld, so the stress concentration in the inner weld is greater than the stress concentration in the outer weld.

## 6. Conclusions

Drawing upon the formation mechanism and distribution pattern of residual stress in the inner and outer wall welds of pipelines, this paper constructs a magnetic charge theoretical model tailored for these welds. Subsequently, it analyzes and calculates the weak magnetic signals and their amplitude growth rates in the inner and outer wall welds under varying excitation intensities.

Differences exist in the stress distribution between the inner and outer wall welds. The stress peak value of the outer wall welds is smaller than that of the inner wall welds, and the stress concentration range is wider in comparison. These variations lead to differences in the weak magnetic signals of the inner and outer wall welds. Specifically, the peak-to-valley values of the normal component and the peak value of the tangential component in the outer wall welds are both lower than those in the inner wall welds. Furthermore, the peak-to-valley width of the normal component and the peak width of the tangential component are both larger than those of the inner wall welds.

The excitation intensity impacts the amplitude of the weak magnetic signal, with its effect gradually diminishing as the excitation intensity increases, following a first-order exponential decline relationship. The average attenuation rate of the normal component amplitude growth rate is 34.03% for the inner wall weld and 31.75% for the outer wall weld. Similarly, the average attenuation rate of the tangential component amplitude growth rate is 27.9% for the inner wall weld and 28.01% for the outer wall weld.

The experimental results demonstrate that weak magnetic detection technology can accurately detect the weak magnetic signals of both inner and outer wall welds, effectively validating the accuracy of the theoretical model. The results can be applied to the weak magnetic internal inspection of pipelines to better assess the quality of weld seams and are of great significance in guiding the identification of internal and external defects present in weld seams.

## Figures and Tables

**Table 1:** Mechanical Properties of X70 High Strength Low Alloy Steel.

Grade	Applicable Standards	YS (MPa)	TS (MPa)	Akv (J)
Pipe (>762~1219)	Q/BQB API SPEC 5L [39]	≥485	≥570	≥27

## Data Availability

For ethical and privacy reasons, research data are not publicly available; please contact the corresponding author if required.
